# Urinary DNA Methylation Profiling Identifies a Biologically Distinct Subgroup of Low-Grade Urothelial Carcinoma with High-Grade-Associated Features

**DOI:** 10.3390/ijms27198934

**Published:** 2026-10-08

**Authors:** Marc Andreu-Inglés, Alina-Iuliana Onoiu, Marc Grifoll-Escoda, Anna Hernández-Aguilera, Josep Gumà-Padró, Francesc Riu, Karla Peña, Jordi Camps, David Parada

**Affiliations:** 1 Department of Pathology, Hospital Universitari Sant Joan, 43204 Reus, Spain; marc.andreu@salutsantjoan.cat (M.A.-I.); anna.hernandez@salutsantjoan.cat (A.H.-A.); francesc.riu@salutsantjoan.cat (F.R.); karlabeatriz.pena@salutsanjoan.cat (K.P.); 2 Unitat de Diagnòstic Molecular, Hospital Universitari Sant Joan, 43204 Reus, Spain; 3 Department of Basic Medical Sciences, Faculty of Medicine, Universitat Rovira I Virgili, 43204 Reus, Spain; alinaiuliana.onoiu@estudiants.urv.cat; 4 Unitat de Recerca Biomèdica, Institut de Recerca Biomèdica de la Catalunya Sud, 43204 Reus, Spain; jorge.camps@salutsantjoan.cat; 5 Institut d’Oncologia de la Catalunya Sud, 43204 Reus, Spain; marc.grifoll@salutsantjoan.cat (M.G.-E.); josep.guma@salutsantjoan.cat (J.G.-P.); 6 Grup d’Investigació Oncològica, Translacional, Epidemiològica i Clínica, Institut de Recerca Biomèdica de la Catalunya Sud, 43204 Reus, Spain; 7 Department of Medicine and Surgery, Faculty of Medicine, Universitat Rovira I Virgili, 43204 Reus, Spain

**Keywords:** bladder cancer, low-grade urothelial carcinoma, DNA methylation, liquid biopsy, epigenetics, tumor heterogeneity, digital pathology, MTAP, Ki67

## Abstract

Bladder cancer surveillance remains challenging because conventional methods do not fully capture the biological heterogeneity within histologically low-grade urothelial carcinoma (LGUC). We evaluated whether urinary DNA methylation profiling using Bladder EpiCheck™ can identify clinically relevant LGUC subgroups in a retrospective single-center cohort of 134 patients with paired histopathology, urine cytology, and methylation results. Methylation positivity increased across negative histopathology, LGUC, and high-grade urothelial carcinoma (HGUC) (18.4%, 51.4%, and 91.5%, respectively). Within LGUC, 19 of 37 tumors (51.4%) were methylation-positive. Their EpiScores overlapped those of HGUC, whereas methylation-negative LGUCs exhibited scores similar to negative histopathology. Gaussian mixture modeling independently supported a bimodal distribution. Methylation-positive LGUCs demonstrated a higher Ki67 labeling index, more frequent MTAP loss (33.3% vs. 11.8%), and more recurrences, including recurrence as HGUC, than methylation-negative LGUCs. Conventional nuclear morphometry did not distinguish between the two LGUC subgroups. These exploratory findings suggest that urinary DNA methylation may identify a biologically distinct subset of histologically low-grade tumors with selected high-grade-associated features. Prospective external validation is required before methylation status can inform surveillance or treatment decisions.

## 1. Introduction

Bladder cancer (BCa) represents a substantial and enduring global health burden. As one of the ten most frequently diagnosed malignancies worldwide, its incidence exhibits considerable geographic variation and continues to rise in parallel with population aging [1,2]. A persistent and pronounced sex disparity is evident, with men experiencing approximately a fourfold higher incidence than women [2,3]. Beyond its epidemiological footprint, BCa is defined by marked biological heterogeneity and a strong propensity for recurrence and progression, making refined risk stratification and sustained surveillance central to contemporary disease management [2,3].

Urothelial carcinoma (UCa) accounts for 90–95% of all BCa cases and encompasses a clinical spectrum ranging from non-muscle-invasive bladder cancer (NMIBC) to muscle-invasive bladder cancer (MIBC) [3,4]. At initial diagnosis, nearly three-quarters of tumors are NMIBC [2,3]. While many of these lesions follow an indolent course, high-risk NMIBC carries a meaningful probability of progression, thereby necessitating stringent follow-up [2]. In contrast, MIBC typically demonstrates aggressive biological behavior and is associated with inferior survival outcomes, requiring accurate staging and timely multimodal management [3,5].

Cystoscopy in combination with urine cytology remains the cornerstone for diagnosis and surveillance [2,3]. Nevertheless, cystoscopy is invasive and costly, whereas urine cytology, despite excellent specificity for high-grade disease, shows limited sensitivity, particularly for low-grade tumors [2,4]. Although advanced imaging modalities, including multiparametric MRI interpreted through the VI-RADS system, have improved the accuracy and reproducibility of non-invasive local staging, histopathological confirmation continues to be mandatory [5,6].

Against this backdrop, liquid biopsy has emerged as a minimally invasive tool enabling real-time assessment of tumor-derived biomarkers [4]. DNA methylation-based assays offer strong biological rationale owing to the early, stable and widespread nature of epigenetic alterations during carcinogenesis [4,7]. Bladder EpiCheck™ is a quantitative urine-based test analyzing 15 methylation markers [7,8]. It has demonstrated high diagnostic performance for detecting high-grade NMIBC recurrence and has received FDA 510(k) clearance for use in NMIBC surveillance alongside cystoscopy [8,9,10]. Notably, the test has shown the capacity to identify high-grade recurrences missed by white-light cystoscopy and to provide anticipatory molecular signals that may enhance clinical decision-making [7,10,11].

This study aimed to determine the diagnostic accuracy of Bladder EpiCheck™ in a real-world cohort undergoing surveillance following transurethral resection of bladder tumor (TURBT). We compared its performance with urine cytology and evaluated its ability to identify biologically aggressive disease beyond conventional histopathological grading. In addition, we assessed the clinical implications of discordant test results and explored whether urinary DNA methylation profiling could improve risk stratification and facilitate more personalized surveillance approaches for patients with urothelial carcinoma [2,7,10].

## 2. Results

### 2.1. Clinical Findings

The final analysis included 134 patients with paired histopathological evaluation, urine cytology, and Bladder EpiCheck™ results. The study cohort was predominantly male, with men accounting for 94.7% (36/38) of patients with negative histopathology, 83.8% (31/37) of those with low-grade urothelial carcinoma (LGUC), and 81.3% (48/59) of those with high-grade urothelial carcinoma (HGUC) (Table 1).

Sex distribution did not differ significantly among diagnostic groups. In contrast, age varied significantly across groups, with patients diagnosed with HGUC being older than those with negative findings or LGUC lesions (Table 1).

Urine cytology findings also differed significantly according to diagnostic category. Among patients with histologically confirmed HGUC, only 17 of 59 (28.8%) were classified as positive for HGUC by cytology (Category V according to the Paris System). The remaining cases were categorized as negative for HGUC (Category II; 10/59, 17.0%), atypical urothelial cells (Category III; 12/59, 20.3%), or suspicious for HGUC (Category IV; 20/59, 33.9%). These findings highlight the limited sensitivity of urine cytology for identifying HGUC when Category V is considered the only positive result. Based on histopathological diagnosis, 38 patients (28.4%) showed no evidence of malignancy (Negative), 37 (27.6%) were diagnosed with LGUC, and 59 (44.0%) with HGUC (Table 1).

### 2.2. Methylation Findings

Methylation positivity increased significantly across histopathological categories, ranging from 18.4% (7/38) in patients with negative histopathology to 51.4% (19/37) in those with LGUC and 91.5% (54/59) in patients with HGUC (Table 1, Figure 1B; *p* < 0.001). Quantitative methylation scores increased stepwise with tumor grade. Median scores were 29 (IQR 17–39) in the negative histopathology group, 62 (IQR 22–74) in the LGUC group, and 90 (IQR 79–95) in the HGUC group (Table 1). Pairwise comparisons confirmed significant differences between all diagnostic categories (all *p* < 0.05).

Among the 38 patients with negative histopathology, 7 (18.4%) had a positive Bladder EpiCheck™ result. We defined “Negative histopathology” as the absence of malignancy in the paired TURBT or biopsy specimen and did not consider it alone proof of an analytical false-positive methylation result. We reviewed each discordant case for specimen adequacy, the interval between urine collection and tissue sampling, contemporaneous cystoscopic and cytological findings, prior urothelial carcinoma, and subsequent histologically confirmed recurrence during follow-up. Because a negative tissue sample may reflect limited sampling or occult disease, we classified cases without a subsequently detected tumor as clinically discordant rather than definitively false positive.

Using the prespecified Bladder EpiCheck™ positivity threshold of 60, diagnostic performance varied by the clinical endpoint evaluated. ROC analysis of continuous EpiScore values was conducted as an exploratory assessment. Youden’s index yielded an estimated cutoff of 74.5 for the evaluated HGUC endpoint (Figure 1C), whereas the estimated cutoff for malignant versus negative histopathology was 60.5 (Figure 1D). These estimates were endpoint- and cohort-specific, were not used for LGUC subgroup assignment, and should not be interpreted as replacements for the validated threshold of 60. For detection of HGUC versus all other cases (LGUC and Negative), sensitivity was 91.5%, specificity was 65.3%, PPV was 67.5%, and NPV was 90.7%. For identification of any urothelial carcinoma (LGUC + HGUC) versus Negative cases, sensitivity and specificity were 76.0% and 81.6%, respectively, with a PPV of 91.3% and an NPV of 57.4%. When the analysis was restricted to HGUC versus Negative cases, sensitivity remained 91.5%, while specificity increased to 81.6%, yielding a PPV of 88.5% and an NPV of 86.1%.

### 2.3. Correlation with Methylation Testing and Histopathology

Methylation analysis identified substantial molecular heterogeneity within histologically defined LGUCs. While LGUC is conventionally regarded as a single pathological entity, methylation scores segregated these tumors into two distinct subgroups (Figure 2A). One subgroup exhibited low methylation scores overlapping those of histologically negative cases, whereas the second displayed high methylation scores comparable to those observed in HGUC (Figure 2B). Accordingly, methylation-negative LGUCs were indistinguishable from Negative cases, while methylation-positive LGUCs showed methylation profiles that were statistically indistinguishable from HGUCs. Unsupervised Gaussian mixture modeling independently confirmed two discrete clusters, providing quantitative support for a bimodal methylation pattern within LGUC. These findings suggest that methylation testing captures biological heterogeneity not apparent from conventional histopathological grading alone.

### 2.4. Morphometric Analysis

Quantitative digital pathology revealed a progressive increase in nuclear size with increasing histological grade (Figure 3C, Table 2). Compared with negative histopathology, both methylation-negative and methylation-positive LGUCs exhibited significantly larger nuclear areas, while HGUCs demonstrated the highest nuclear area values overall. Notably, nuclear area did not differ significantly between the two LGUC methylation subgroups, indicating that the molecular heterogeneity identified by methylation profiling was not accompanied by detectable differences in this conventional morphometric parameter (Figure 3C, Table 2).

In contrast, cellular density remained comparable across all diagnostic categories and showed no significant association with either histological grade or methylation status (Table 2). These findings suggest that nuclear enlargement reflects increasing histological severity, whereas digital morphometric features alone do not discriminate between the molecularly distinct LGUC subgroups identified by methylation analysis.

### 2.5. Immunohistochemical Analysis

We evaluated HER2, GATA3, and CK5/6 expression in 17 methylation-negative LGUCs, 18 methylation-positive LGUCs, and HGUCs. HER2 showed heterogeneous staining intensities, ranging from 0+ (including umbrella cell-restricted staining) to 3+, with no significant differences in expression-pattern distribution among groups (Supplementary Table S1). GATA3 expression was largely retained across all evaluable tumors, with nearly all cases demonstrating strong (3+) staining and only isolated cases showing moderately reduced (2+) expression. CK5/6 also exhibited variable staining patterns including absent, basal-restricted, and diffuse 1+ to 3+ expression—but did not differ significantly among diagnostic groups (Supplementary Table S1).

In contrast, MTAP loss was more frequent in methylation-positive than in methylation-negative LGUCs. Loss of MTAP expression was observed in 2 of 17 methylation-negative LGUCs (11.8%) and 6 of 18 methylation-positive LGUCs (33.3%). A comparable frequency of MTAP loss was identified in HGUCs (12/35, 34.3%) (Figure 3E).

Digital image analysis further demonstrated significant group-level differences in the mean nuclear area of Ki67- and p53-positive tumor cells (Supplementary Table S2). For both markers, positive nuclei were significantly larger in HGUCs than in either methylation-defined LGUC subgroup. No significant differences in the nuclear area of Ki67- or p53-positive cells were detected between methylation-negative and methylation-positive LGUCs. The proportion of p53-positive tumor cells did not differ significantly among groups, despite the presence of both wild-type and overexpression staining patterns (Figure 3B). By contrast, the Ki67 labeling index increased progressively across the diagnostic spectrum and differed significantly among all groups. Notably, methylation-positive LGUCs exhibited a significantly higher Ki67 labeling index than methylation-negative LGUCs, indicating greater proliferative activity within the methylation-positive subgroup (Figure 3A–D).

### 2.6. Clinical Follow-Up Evaluation

During follow-up, histologically confirmed recurrence was more common among patients with methylation-positive LGUC than among those with methylation-negative LGUC (Figure 3F). In the methylation-positive subgroup, 21.1% developed recurrent LGUC and 15.8% developed recurrent HGUC. By contrast, 5.9% of patients with methylation-negative LGUC developed recurrent LGUC, and none developed histologically confirmed HGUC. We did not count cystoscopic, cytological, or molecular abnormalities without tissue confirmation as recurrence events.

### 2.7. Multivariate Integration of Methylation, Proliferation and MTAP Expression

We conducted FAMD as an exploratory complete-case analysis. We excluded negative-histopathology cases because tumor-based Ki67 and MTAP measurements were not applicable. Of 96 patients with LGUC or HGUC, 45 had complete data for all three prespecified variables (EpiScore, Ki67 labeling index, and dichotomized MTAP expression) and were included. We excluded the remaining 51 tumor cases because at least one tissue-based biomarker measurement was unavailable or not evaluable; we did not impute missing values. The first two dimensions accounted for 83.7% of total inertia, with Dimension 1 explaining 55.8% and Dimension 2 27.9%. Methylation score and Ki67 were the main contributors to Dimension 1 (42.6% and 35.0%, respectively), whereas MTAP was the main contributor to Dimension 2 (70.5%). Dimension 1 scores differed among groups and showed significant differences for all three comparisons (Figure 4).

## 3. Discussion

This study shows that urinary DNA methylation analysis provides clinically relevant information beyond its established diagnostic application. The diagnostic performance observed in our cohort is consistent with the biological basis of DNA methylation biomarkers [12,13,14]. Aberrant promoter methylation is an early event in urothelial carcinogenesis and may remain relatively stable throughout tumor evolution [15,16,17,18]. Bladder EpiCheck™ showed high sensitivity and negative predictive value for detecting high-grade urothelial carcinoma (HGUC), consistent with previous studies and supporting its use as a non-invasive adjunct to conventional surveillance strategies [9,10].

The threshold of 60 was retained because it aligns with the commercial assay’s prespecified interpretation rule and enables comparison with prior validation studies. Although ROC analysis identified a higher cohort-specific cutoff for one HGUC endpoint, optimizing the threshold in the same cohort used to evaluate performance may inflate apparent accuracy and reduce transportability. The ROC-derived values should be regarded as exploratory estimates rather than clinically actionable alternatives. Reclassification using a higher cutoff would likely reduce the number of methylation-positive LGUCs and may increase specificity at the expense of sensitivity. However, the clinical consequences cannot be established without external validation and decision-analytic assessment.

Beyond diagnostic performance, the study identified two biologically and clinically distinct subgroups within histologically defined low-grade urothelial carcinoma (LGUC). Methylation-positive LGUCs exhibited molecular, pathological, and clinical features more closely resembling those of HGUC than those of methylation-negative LGUC. These findings suggest that urinary DNA methylation captures biologically relevant heterogeneity that is not fully resolved by conventional histopathological assessment.

Histopathological diagnosis and grading remain central to bladder cancer risk stratification. Nevertheless, accumulating molecular evidence indicates that tumors with similar morphological features may harbor substantially different genomic and epigenomic profiles [19]. The bimodal distribution of methylation scores among LGUCs in our cohort reinforces this concept. This distribution suggests that histologically low-grade disease may encompass biologically distinct entities rather than a single homogeneous clinicopathological category [20]. Methylation-negative LGUCs showed score distributions that largely overlapped those of histologically negative biopsies, whereas methylation-positive LGUCs displayed scores approaching those observed in HGUC. Gaussian mixture modeling independently reproduced this separation, reducing the likelihood that the observed distribution resulted from arbitrary subgrouping or statistical variation.

Methylation positivity was observed in 18.4% of patients with negative contemporaneous histopathology. This finding should not be interpreted as an equivalent analytical false-positive rate, because a negative biopsy or TURBT specimen may not exclude occult or unsampled disease given urothelial multifocality and field cancerization. Other potential explanations include residual methylation signals after prior treatment or molecular alterations that precede morphological detection. Consistent with this interpretation, a prospective multicenter study reported an overall specificity of 88.0% and a positive predictive value of 44.8% for Bladder EpiCheck [9], with lower sensitivity for low-grade tumors than for high-grade disease. However, these estimates cannot be directly extrapolated to our cohort because test performance, particularly predictive values, depends on disease prevalence, cohort composition, and the reference standard. Therefore, methylation-positive cases with negative histopathology were considered clinically discordant rather than definitively false positive. Standardized longitudinal follow-up with repeated sampling and histopathological confirmation is required to determine whether this pattern reflects analytical false positivity, occult disease, or anticipatory molecular detection.

The pathological findings further supported the distinction between methylation-defined LGUC subgroups. Nuclear area increased progressively across the histological spectrum, from negative histopathology to LGUC and HGUC. However, neither quantitative morphometry nor conventional histopathological evaluation distinguished methylation-positive from methylation-negative LGUC. Thus, the methylation-defined subgroups are unlikely to reflect differences detectable using conventional morphological assessment alone. Instead, they may represent epigenetically distinct tumor states that retain similar low-grade architecture and cytological features.

In contrast to the morphometric findings, Ki67 expression differed significantly between the two LGUC subgroups, with methylation-positive tumors demonstrating greater proliferative activity. Ki67 is an established marker of cellular proliferation and has been associated with recurrence and progression in urothelial carcinoma [20,21,22]. The higher Ki67 labeling index observed in methylation-positive LGUC therefore suggests that the epigenetic alterations detected by Bladder EpiCheck™ are associated with increased proliferative potential despite the preservation of low-grade histological morphology.

The discordance between morphology and proliferative activity may reflect the different biological dimensions these approaches capture. Histopathological grading primarily evaluates architectural organization and cytological atypia. In contrast, methylation profiling measures molecular alterations that may arise early in tumor development, before overt morphological progression becomes apparent [23,24,25,26]. Although the temporal relationship between epigenetic dysregulation and histological transformation remains incompletely understood, methylation-positive LGUCs may have acquired molecular programs associated with more aggressive behavior before developing the architectural and cytological features required for classification as HGUC. Longitudinal molecular studies will be necessary to determine whether methylation positivity represents an early stage of biological progression or a stable molecular subtype of LGUC.

Differences in MTAP expression provided additional evidence of biological divergence between the LGUC subgroups. MTAP loss was more frequent in methylation-positive than in methylation-negative LGUC and occurred at a frequency comparable to that observed in HGUC. MTAP immunohistochemical loss has emerged as a potential surrogate for homozygous deletion of chromosome 9p21.3, a region that includes MTAP and the adjacent CDKN2A locus. Alterations involving this region have been associated with aggressive pathological features in urothelial carcinoma [27,28]. Genomic testing was not performed, and MTAP loss alone cannot establish the presence or extent of a 9p21.3 deletion. Nevertheless, the association of MTAP loss with methylation positivity and increased proliferative activity supports the presence of a biologically distinct LGUC subgroup with selected HGUC-like molecular features.

Factor Analysis of Mixed Data (FAMD) was performed as an exploratory multivariate analysis to jointly assess Methylation Score, Ki67 labeling index, and dichotomous MTAP expression status in cases with complete data for all three variables. Methylation-negative LGUCs, methylation-positive LGUCs and HGUCs showed a significant progressive separation along the first factorial dimension, with methylation-positive LGUCs occupying an intermediate position between methylation-negative LGUCs and HGUCs. These findings suggest that the methylation-defined LGUC subgroups are not distinguished by methylation status alone. Instead, they exhibit different multivariate biological profiles that also incorporate proliferative activity and MTAP expression.

Clinical outcomes further reinforced the relevance of the methylation-defined classification. Recurrence was more frequent among patients with methylation-positive LGUC, and recurrence as HGUC occurred exclusively in this subgroup. In contrast, recurrence was uncommon among patients with methylation-negative LGUC, and no high-grade recurrences were observed during follow-up. These findings suggest that methylation positivity may identify a subset of histologically low-grade tumors with a less favorable clinical course. However, because of the limited sample size and number of outcome events, the prognostic magnitude of this association should be interpreted cautiously.

If independently validated, urinary methylation profiling could complement established clinicopathological risk stratification models [29]. Patients with methylation-positive LGUC might benefit from closer surveillance despite having low-grade histology, whereas methylation-negative status could potentially identify patients suitable for less intensive follow-up. Such an approach could help align surveillance intensity with underlying tumor biology rather than morphology alone. Nevertheless, treatment or surveillance strategies should not be modified based on these findings until their prognostic value and clinical utility have been confirmed prospectively.

The present study has several limitations. First, this was a single-center study that included a relatively small number of methylation-defined LGUCs, particularly for the analyses of overall and high-grade recurrence. Importantly, the methylation-defined subgroups were identified and characterized using pathomorphological, immunohistochemical, and clinical variables within the same cohort. This internally derived approach introduces a risk of overfitting and may overestimate the reproducibility and prognostic relevance of the proposed classification. Because no independent validation cohort was available, the observed subgroup differences should be regarded as exploratory and hypothesis-generating. External validation in larger, prospectively assembled, multicenter cohorts with prespecified analytical procedures and standardized longitudinal follow-up is required before the classification can be considered robust or clinically applicable. Second, follow-up was insufficient to evaluate long-term progression, disease-specific survival, or overall survival. Third, comprehensive genomic and epigenomic characterization was unavailable, precluding direct correlation of methylation status with underlying molecular alterations, including MTAP and CDKN2A deletions. Fourth, the methylation-based LGUC subgroups were identified and characterized within the same cohort, which may introduce a risk of overfitting and limit the generalizability of the observed pathomorphological and clinical associations. Therefore, the proposed classification and its associated findings should be considered exploratory and hypothesis-generating. Validation in larger, independent, preferably multicentric cohorts are required. Fifth, the exploratory FAMD was limited to 45 complete cases and may therefore be affected by selection bias if tissue availability or evaluability was associated with tumor characteristics. These multivariate clustering findings should be considered hypothesis-generating and require validation in a larger cohort with prospectively complete biomarker assessment. Finally, the observational design does not establish whether methylation-guided surveillance improves outcomes, reduces cystoscopy burden or is cost-effective.

In conclusion, urinary DNA methylation profiling identified exploratory biological heterogeneity within histologically defined LGUC that conventional morphology did not capture. Within this internally derived cohort, methylation-positive LGUCs showed greater proliferative activity, more frequent MTAP loss, and more recurrences, including recurrence as high-grade disease. These associations support the hypothesis that a subset of histologically low-grade tumors may harbor selected high-grade-associated features. However, the proposed classification has not been independently validated and should not be used to guide surveillance or treatment. Larger prospective multicenter studies with prespecified thresholds, standardized follow-up, and integrated genomic characterization are required to assess reproducibility, prognostic value, and clinical utility.

## 4. Materials and Methods

### 4.1. Study Design and Patient Selection

This study was designed as a single-center observational cohort investigation including patients undergoing routine surveillance for bladder cancer during surveillance after transurethral resection of bladder tumor (TURBT). We screened consecutive patients evaluated between January 2020 and January 2026 for eligibility.

Inclusion criteria comprised patients with a clinical or cystoscopic suspicion of urothelial carcinoma of the bladder who had available histopathological results from TURBT or biopsy and paired urine cytology and Bladder EpiCheck™ testing. We included patients with both malignant and non-malignant histopathological outcomes.

Patients were excluded if Bladder EpiCheck™ testing was performed more than six months after the corresponding biopsy or if clinical, cytological, or histopathological data were incomplete.

### 4.2. Ethical Considerations

The study was conducted in accordance with the Declaration of Helsinki and applicable national and European regulations governing biomedical research. The study protocol, including its first amendment, as well as the informed consent form and patient information sheet, received approval from the Comitè Ètic d’Investigació amb Medicaments (CEIm) de l’Institut d’Investigació Sanitària Pere Virgili (reference CEIm: 099/2024), with a favorable opinion issued on 27 February 2025.

All data were handled confidentially and anonymized prior to analysis to protect participant privacy and ensure compliance with current data protection legislation. As this was an observational study conducted within routine clinical practice, participants were not exposed to any additional risks or interventions beyond standard care.

### 4.3. Clinical Evaluation and Follow-Up

All patients received standard monitoring in line with current European Association of Urology guidelines [3,30], which included cystoscopy and urine cytology. Biopsies or repeat TURBT were performed when clinically indicated. Clinical data collected included age, gender, smoking habits, tumor stage and histological grade at initial diagnosis, as well as previous treatments.

We calculated clinical follow-up from the date of the index histopathological diagnosis associated with the paired urine methylation assessment to the date of the last available urological evaluation or histologically confirmed recurrence. Recurrence was defined as a newly detected urothelial carcinoma confirmed by histopathological examination of a subsequent biopsy or TURBT specimen after the index evaluation. We classified recurrences by the highest histological grade at the time of recurrence as LGUC or HGUC. Abnormal cystoscopic, cytological, imaging, or urinary molecular findings without histopathological confirmation were not counted as recurrence events. Patients without histologically confirmed recurrence were censored at their last documented follow-up.

All biopsy specimens were formalin-fixed, paraffin-embedded, and processed using standardized histopathological protocols. Histological diagnosis and tumor grading were established according to the 2022 WHO Classification of Tumours of the Urinary and Male Genital Systems (5th edition). Cases were categorized as negative for malignancy, non-muscle-invasive bladder cancer (NMIBC), or muscle-invasive bladder cancer (MIBC). Urothelial carcinomas were further stratified into low-grade and high-grade lesions based on WHO-defined morphological criteria [31].

### 4.4. Bladder Epicheck Assay

For the Bladder EpiCheck™ test (Nucleix Ltd, Rehovo, Israel), urine samples were centrifuged twice at 1000× *g* for 5 min at room temperature. DNA was isolated using the Bladder EpiCheck™ DNA extraction kit and treated with a methylation-sensitive restriction enzyme that cleaves DNA at its recognition sites when unmethylated.

Samples were then prepared for polymerase chain reaction (PCR) analysis using the Bladder EpiCheck™ test kit, and results were evaluated using the Bladder EpiCheck™ software (version 1.9). For samples that passed internal control validation, the Bladder EpiCheck™ software generated an EpiScore ranging from 0 to 100. An EpiScore ≥ 60 was classified as positive, and an EpiScore < 60 as negative, consistent with the prespecified commercial threshold and previously published validation studies [9] (Figure 1A). We retained this threshold for all primary categorical and LGUC subgroup analyses to avoid cohort-specific post hoc optimization and to preserve comparability with established test interpretation.

### 4.5. Urine Cytology

Urine cytology was performed on samples collected during the same surveillance visit as Bladder EpiCheck™ testing using the Papanicolaou staining procedure [32]. Cytological findings were classified as negative, atypical, suspicious, or positive for malignancy according to the Paris System for Reporting Urinary Cytology [33].

### 4.6. Histopathological and Immunohistochemical Analysis

Bladder tissue biopsies were obtained during transurethral resection of bladder tumor (TURBT) procedures and processed according to standardized institutional protocols. Specimens were fixed in formalin and subsequently embedded in paraffin. Paraffin blocks were sectioned at a thickness of 4 µm and prepared for routine microscopic evaluation. Histopathological assessment was performed on hematoxylin and eosin (H&E)-stained slides using validated automated hospital protocols.

Protein biomarker expression was evaluated by immunohistochemistry (IHC) using the fully automated Ventana BenchMark ULTRA platform (Roche Diagnostics, Indianapolis, IN, USA). The antibody panel included Ki67, p53, GATA3, HER2, CK5/6, and MTAP. Immunostaining was performed using either the OptiView DAB IHC Detection Kit or the ultraView Universal DAB Detection Kit (Roche, Spain), according to the specific requirements of each antibody. Signals were visualized using diaminobenzidine (DAB) chromogen, followed by hematoxylin counterstaining. Quality control included internal and external positive controls in each staining run. Detailed staining protocols are provided in Supplementary Table S3.

### 4.7. Digital Pathology

A Ventana DP 200 slide scanner (Roche, Basel, Switzerland) was used to digitize slides at 40× magnification. Image analysis was performed using QuPath software (version 0.5.1). A project was created and whole-slide images were imported, with the default H-DAB filter selected for immunohistochemical image analysis. To ensure accurate color representation, the “Estimate Stain Vectors” function was applied to each image for automatic color-scale correction.

Tissue annotations were created manually to delineate tissue sections as regions of interest (ROIs) and categorize them as tissue areas. To exclude non-representative or damaged peripheral regions, the “Expand Annotations” function was applied with an expansion radius of −100 μm. Artifacts within tissue sections were manually corrected by erasing them or adjusting the annotation regions as required.

Two primary analysis tools were used according to the observed staining patterns. The “Cell Detection” tool was used for discrete cellular markers characterized by well-defined cell boundaries with clear membrane and/or cytoplasmic localization. The “Pixel Classifier” tool was applied to diffuse or continuous staining patterns characterized by overlapping marker distribution that did not conform to discrete cellular boundaries.

Individual cells in H&E-stained LGUC and HGUC sections were identified and quantified using the “Cell Detection” algorithm. The algorithm was configured using “Hematoxylin OD” detection parameters at a pixel resolution of 0.5 μm. Default settings were maintained for all parameters except the intensity threshold, which was adjusted to 0.1.

For antibodies exhibiting diffuse or continuous staining patterns, including Ki67 and p53, the “Pixel Classifier” tool was used. The algorithm was configured using “Diaminobenzidine OD” detection parameters at a pixel resolution of 0.5 μm. Default settings were maintained for all parameters except the intensity threshold, which was adjusted to 0.1. For cellular staining patterns, positivity was quantified as the percentage of immunopositive cell area relative to the total ROI area: (immunopositive cell area/total ROI area) × 100%. For diffuse staining patterns, positivity was expressed as the percentage of DAB-stained area relative to the total ROI area: (DAB-stained area/total ROI area) × 100%. Following pixel classification, tissue objects were systematically generated to enable downstream quantitative analysis.

After cell detection or pixel classification, the resulting tissue objects were enhanced with spatial measurements using the “Analyze > Calculate Features > Add Shape Features” function to incorporate morphometric parameters into the quantitative analysis. Upon completion of the analysis, QuPath generated comprehensive quantitative datasets for each case. All image-associated data and analysis parameters were systematically saved and archived under the respective patient identifiers to ensure data integrity and facilitate subsequent statistical analysis.

### 4.8. Statistical Analysis

Statistical analyses were performed using R version 4.6.1 (R Foundation for Statistical Computing, Vienna, Austria). Continuous variables are presented as median and interquartile range (IQR), whereas categorical variables are reported as frequencies and percentages.

Comparisons between two independent groups were performed using the Wilcoxon rank-sum test. Comparisons involving three or more independent groups were performed using the Kruskal–Wallis test, followed, when appropriate, by pairwise Wilcoxon rank-sum tests with Holm adjustment for multiple comparisons. Categorical variables were compared using Pearson’s chi-square test or Fisher’s exact test, as appropriate.

The diagnostic performance of Bladder EpiCheck™ was evaluated by calculating sensitivity, specificity, positive predictive value, and negative predictive value. We conducted ROC curve analysis as a secondary exploratory analysis to assess the discrimination of continuous EpiScore values for prespecified histopathological endpoints. We estimated optimal cutoffs using Youden’s index. We did not use these cohort-derived cutoffs to redefine methylation-positive and methylation-negative groups, nor did we consider them clinically validated thresholds.

Factor Analysis of Mixed Data (FAMD) was prespecified as an exploratory complete-case analysis that integrated EpiScore, the Ki67 labeling index, and dichotomized MTAP expression. Eligibility required histologically confirmed LGUC or HGUC and non-missing, evaluable results for all three variables. We excluded patients with negative histopathology because tumor-based Ki67 and MTAP measurements were not applicable. We excluded cases with missing or non-evaluable Ki67 or MTAP results and performed no imputation. The contributions of individual variables to each factorial dimension were assessed, and differences in factorial dimension scores between study groups were evaluated using the Kruskal–Wallis test followed by pairwise Wilcoxon rank-sum tests with Holm adjustment.

All statistical tests were two-sided, and *p* < 0.05 was considered statistically significant.

## Figures and Tables

**Figure 1:**
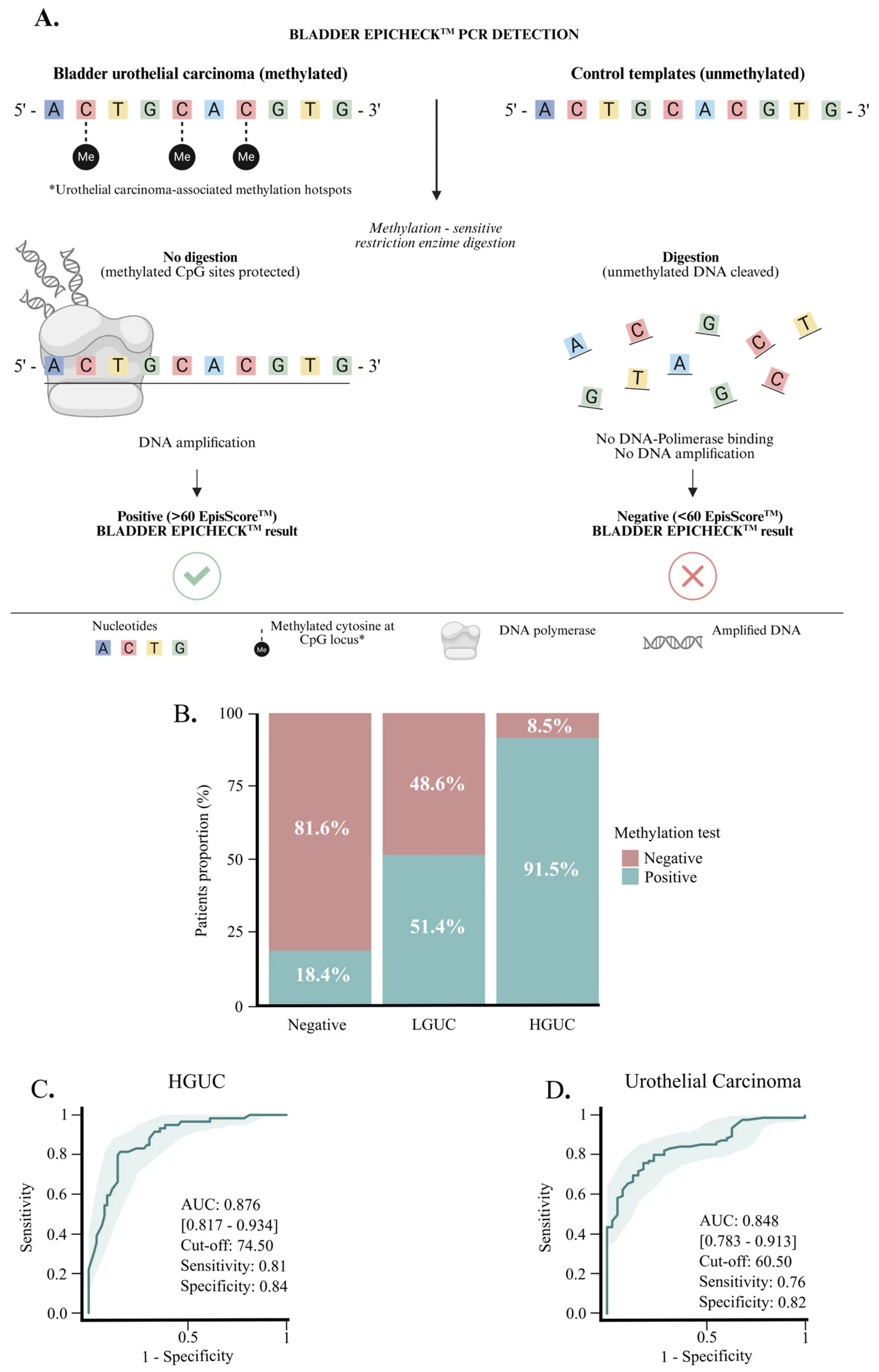
Principle of the Bladder EpiCheck™ assay and diagnostic performance of urinary DNA methylation profiling. (**A**) Schematic representation of the Bladder EpiCheck™ assay. Methylated DNA is protected from digestion by a methylation-sensitive restriction enzyme and selectively amplified by real-time PCR, whereas unmethylated DNA is enzymatically digested and not amplified. (**B**) Distribution of methylation-positive (EpiScore ≥ 60) and methylation-negative (EpiScore < 60) results among patients with negative histopathology, low-grade urothelial carcinoma (LGUC), and high-grade urothelial carcinoma (HGUC). (**C**) Receiver operating characteristic (ROC) analysis of continuous EpiScore values for detection of HGUC versus LGUC + negative histopathology. (**D**) ROC analysis for detection of urothelial carcinoma (LGUC + HGUC) versus negative histopathology. AUC, area under the ROC curve; CI, confidence interval. Report the verified AUC and 95% CI in each panel.

**Figure 2:**
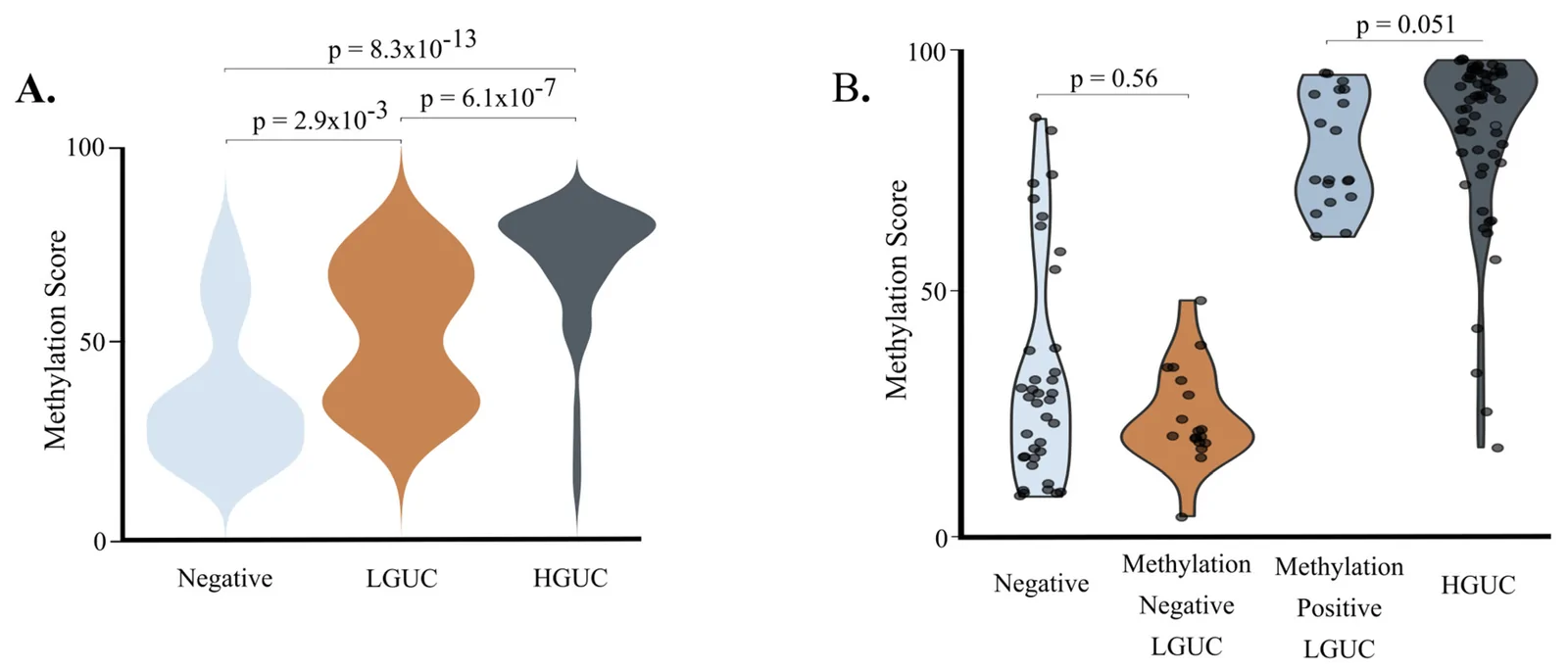
Bladder EpiCheck™ EpiScores identify two distinct exploratory subgroups within LGUC. (**A**) Distribution of EpiScores across negative histopathology, low-grade urothelial carcinoma (LGUC), and high-grade urothelial carcinoma (HGUC). Pairwise comparisons were performed using Wilcoxon rank-sum tests with Benjamini–Hochberg adjustment for multiple comparisons. *p*-values are reported as indicated in the figure. (**B**) We stratified LGUC using the prespecified threshold of 60 into methylation-negative (EpiScore < 60) and methylation-positive (EpiScore ≥ 60) groups. The horizontal dashed line indicates the prespecified positivity threshold. Boxplots show the median and interquartile range; points represent individual patients. LGUC, low-grade urothelial carcinoma; HGUC, high-grade urothelial carcinoma. Comparisons of methylation-negative LGUC with negative histopathology and methylation-positive LGUC with HGUC were performed using Wilcoxon rank-sum tests. *p*-values are reported as indicated in the figure.

**Figure 3:**
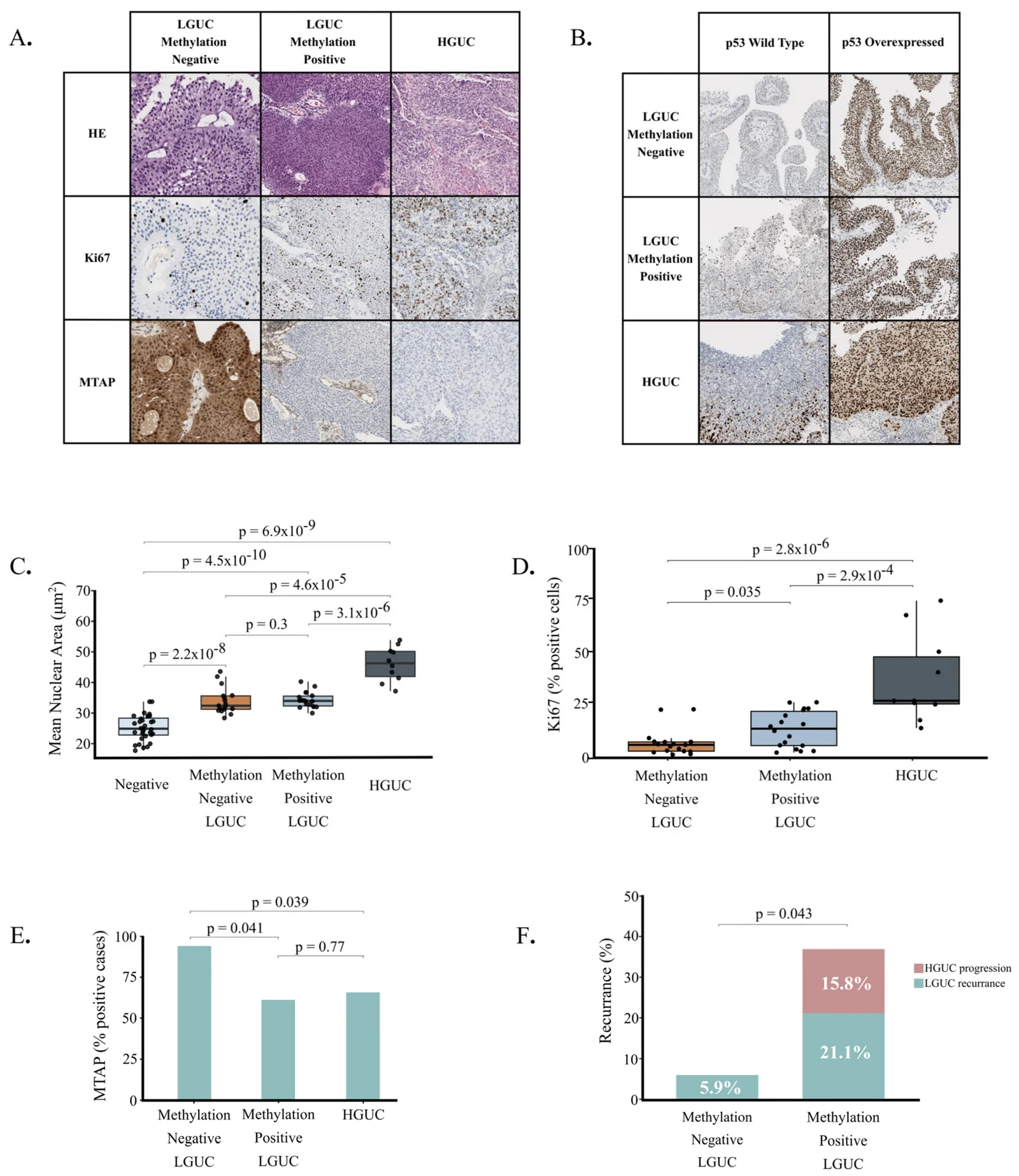
Biological and clinical characteristics of methylation-defined LGUC subgroups. (**A**) Representative hematoxylin and eosin (H&E), Ki67, and MTAP staining in methylation-negative LGUC, methylation-positive LGUC, and HGUC. (**B**) Representative p53 immunohistochemical patterns. (**C**) Nuclear area across diagnostic and methylation-defined groups. Comparisons were performed using Kruskal–Wallis and Holm-adjusted pairwise Wilcoxon tests. (**D**) Ki67 labeling index quantified by digital image analysis. Values were compared using the Wilcoxon rank-sum test. (**E**) MTAP expression quantified by immunohistochemistry. Categories were compared using Fisher’s exact test. (**F**) Histologically confirmed clinical outcomes during available follow-up, categorized as recurrent LGUC, recurrent HGUC, or no confirmed recurrence. Categories were compared using Fisher’s exact test. Statistical significance is indicated in the corresponding panels. Boxplots display medians and interquartile ranges, with points representing individual cases. LGUC, low-grade urothelial carcinoma; HGUC, high-grade urothelial carcinoma; MTAP, methylthioadenosine phosphorylase.

**Figure 4:**
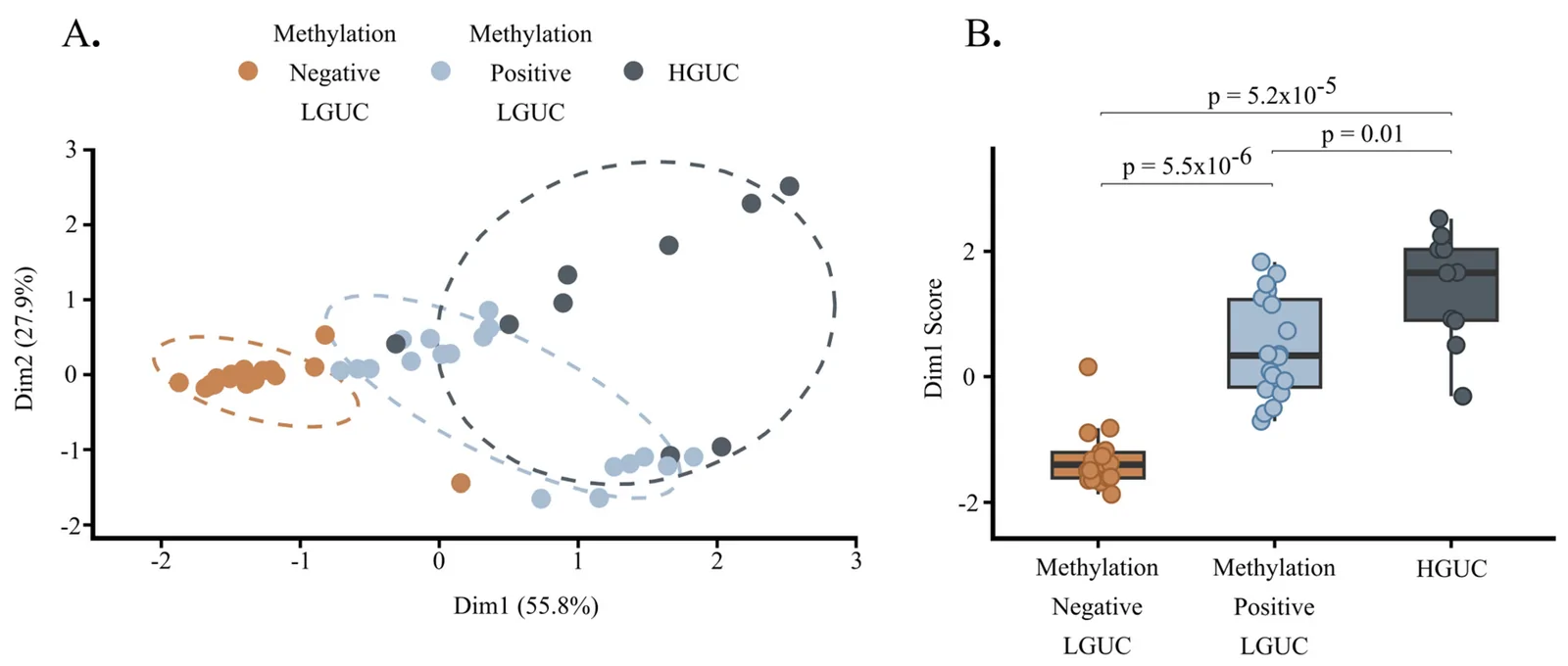
Exploratory Factor Analysis of Mixed Data (FAMD) integrating urinary EpiScore, Ki67 labeling index, MTAP expression, and histological group. (**A**) Factorial map of 45 complete cases with LGUC or HGUC and evaluable data for all three variables. Points represent individual patients; dashed ellipses indicate 68% confidence regions. Dimension 1 explains 55.8% of total inertia and Dimension 2 explains 27.9%. (**B**) Distribution of Dimension 1 scores across methylation-negative LGUC, methylation-positive LGUC, and HGUC. We tested overall differences using the Kruskal–Wallis test, followed by pairwise Wilcoxon rank-sum tests with Holm adjustment. Boxplots show medians and interquartile ranges. FAMD, Factor Analysis of Mixed Data; LGUC, low-grade urothelial carcinoma; HGUC, high-grade urothelial carcinoma; MTAP, methylthioadenosine phosphorylase.

**Table 1:** Clinicopathological, cytological, and methylation characteristics of the study cohort according to histopathological diagnosis.

	Negative *n* = 38	LGUC *n* = 37	HGUC *n* = 59
**Male sex, *n* (%)**	36 (94.7)	31 (83.8)	48 (81.3)
**Age, years ^b,c^**	67 [60–75]	71 [61–77]	75 [68–79]
**Cytology (*Paris System*), *n* (%) ^a,b,c^**			
Unsatisfactory (*Paris I*)	10 (26.3)	0	0
Negative for HGUC (*Paris II*)	14 (36.8)	22 (59.5)	10 (17.0)
Atypical urothelial cells (*Paris III*)	8 (21.0)	12 (32.4)	12 (20.3)
Suspicious for HGUC (*Paris IV*)	6 (15.9)	3 (8.1)	20 (33.9)
Positive HGUC (*Paris V*)	0	0	17 (28.8)
**Invasion, *n* (%) ^b,c^**			
Without	38 (100.0)	37 (100.0)	20 (34.0)
Connective tissue	0	0	26 (44.0)
Muscle	0	0	13 (22.0)
**Methylation test, *n* (%) ^a,b,c^**			
Negative	31 (81.6)	18 (48.6)	5 (8.5)
Positive	7 (18.4)	19 (51.4)	54 (91.5)
**Methylation score ^a,b,c^**	29 [17–39]	62 [22–74]	90 [79–95]

Data are presented as *n* (%) or median (Q1–Q3). Sex distribution and overall Bladder EpiCheck positivity were compared using Pearson’s chi-square test. Age was compared using the Kruskal–Wallis test, followed by pairwise Wilcoxon rank-sum tests with Benjamini–Hochberg adjustment. Pairwise comparisons of proportions for Bladder EpiCheck positivity were performed using pairwise proportion tests with Bonferroni adjustment. Methylation scores were compared using Wilcoxon rank-sum tests with Benjamini–Hochberg adjustment. Adjusted *p*-values are reported where applicable. Significant differences (*p*-value < 0.05) in comparisons are indicated by ^a^—negative histopathology vs. LGUC, ^b^—negative histopathology vs. HGUC, and ^c^—LGUC vs. HGUC.

**Table 2:** Quantitative digital pathology features across histopathological categories and methylation-defined low-grade urothelial carcinoma subgroups.

	Cell Density (cells/mm^2^)	Mean Nuclear Area (µm^2^)
**Negative histopathology**	7326.8 [5010.9–8658.6]	24.9 [22.8–28.3]
**LGUC**	5218.1 [4753.4–5858.0]	33.8 [31.9–35.6]
Methylation Negative LGUC	5161.2 [4841.3–5522.7]	32.4 [31.2–35.6]
Methylation Positive LGUC	5410.1 [4693.3–6169.9]	34.0 [32.3–35.5]
**HGUC**	4847.2 [4251.8–5202.3]	46.3 [41.9–50.2]

Values are reported as the median [interquartile range]. We compared overall groups using the Kruskal–Wallis test, followed by pairwise Wilcoxon rank-sum tests with Holm adjustment. Cell density is expressed as cells/mm^2^, and mean nuclear area as μm^2^. LGUC, low-grade urothelial carcinoma; HGUC, high-grade urothelial carcinoma.

## Data Availability

The data supporting the findings of this study are available from the corresponding author upon reasonable request. Due to privacy and ethical restrictions, individual-level patient data are not publicly available.

## Supplementary Materials

The following supporting information can be downloaded at: https://www.mdpi.com/article/10.3390/ijms27198934/s1.

## Abbreviations

The following abbreviations are used in this manuscript:

BCa	Bladder Cancer
UCa	Urothelial Carcinoma
DNA	Deoxyribonucleic Acid
NMIBC	Non-muscle-invasive Bladder Cancer
MIBC	Muscle-invasive Bladder Cancer
TURBT	Transurethral Resection of Bladder Tumor
LGUC	Low-grade Urothelial Carcinoma
HGUC	High-grade Urothelial Carcinoma
Bx	Biopsy
PPV	Positive Predictive Value
NPV	Negative Predictive Value
ROC	Receiver Operating Characteristic
AUC	Area Under the Curve
FAMD	Factor Analysis of Mixed Data
IQR	Interquartile Range
ROI	Region of Interest
IHC	Immunohistochemistry
MTAP	Methylthioadenosine phosphorylase
