# Supplementary material for: Urinary DNA Methylation Profiling Identifies a Biologically Distinct Subgroup of Low-Grade Urothelial Carcinoma with High-Grade-Associated Features
Source: Int J Mol Sci. 2026 Oct 8;27(19):8934. doi: 10.3390/ijms27198934 (PMC13665559; doi:10.3390/ijms27198934)
Supplement: Supplementary file 1 [file ijms-27-08934-s001.zip › ijms-4558187-supplementary.pdf]

Article

Urinary DNA Methylation Profiling Identifies a Biologically Distinct Subgroup of Low-grade Urothelial Carcinoma with High-grade-associated Features

Marc Andreu-Inglés [1,2,3,4], Alina-Iuliana Onoiu [3,4] , Marc Grifoll-Escoda [5,6], Anna Hernández-Aguilera [1,2,3,4], Josep Gumà-Padró [5,6], Francesc Riu [1,2,3,6], Karla Peña [1,2,3,4] , Jordi Camps [4,7], and David Parada [1,2,3,4,6]*

[1] Department of Pathology, Hospital Universitari Sant Joan, Reus, Spain
[2] Unitat de Diagnòstic Molecular, Hospital Universitari Sant Joan, Reus, Spain
[3] Department of Basic Medical Sciences, Faculty of Medicine, Universitat Rovira I Virgili, Reus, Spain
[4] Unitat de Recerca Biomèdica, Institut de Recerca Biomèdica de la Catalunya Sud, Reus, Spain
[5] Institut d'Oncologia de la Catalunya Sud, Reus, Spain
[6] Grup d'Investigació Oncològica, Translacional, Epidemiològica i Clínica, Institut de Recerca Biomèdica de la Catalunya Sud, Reus, Spain
[7] Department of Medicine and Surgery, Faculty of Medicine, Universitat Rovira I Virgili, Reus, Spain
* Correspondence: david.parada@urv.cat

Supplementary Materials: The following supporting information is available online: **Table S1**. Immunohistochemical profiles of methylation-defined low-grade urothelial carcinoma subgroups and high-grade urothelial carcinoma. **Table S2.** Digital pathology-derived quantification of proliferative and p53-associated biomarkers according to methylation-defined tumor subgroup. **Table S3.** Standardized immunohistochemical protocols for biomarker evaluation using the Ventana BenchMark ULTRA system.

Table S1. Immunohistochemical profiles of methylation-defined low-grade urothelial carcinoma-subgroups and high-grade urothelial carcinoma

Marker	Category	Methylation Negative LGUC	Methylation Positive LGUC	HGUC
**HER2**	**0+ Umbrella**	4/17 (23.5%)	3/18 (16.7%)	0/35 (0.0%)
	**0+**	12/17 (70.6%)	9/18 (50.0%)	18/35 (50.0%)
	**1+**	1/17 (5.9%)	4/18 (22.2%)	7/35 (20.0%)
	**2+**	0/17 (0.0%)	2/18 (11.1%)	0/35 (0.0%)
	**3+**	0/17 (0.0%)	0/18 (0.0%)	11/35 (30.0%)
**GATA3**	**2+**	0/17 (0.0%)	0/18 (0.0%)	4/35 (10.0%)
	**3+**	17/17 (100%)	18/18 (100%)	32/35 (90.0%)
**CK5/6**	**0+**	1/17 (5.9%)	2/18 (11.1%)	7/35 (20.0%)
	**1+**	0/17 (0.0%)	2/18 (11.1%)	4/35 (10.0%)
	**2+**	2/17 (11.8%)	2/18 (11.1%)	0/35 (0.0%)
	**3+**	4/17 (23.5%)	1/18 (5.6%)	11/35 (30.0%)
	**Basal Cells**	10/17 (58.8%)	11/18 (61.1%)	14/35 (40.0%)
**MTAP**	**Positive**	15/17 (88.2%)	12/18 (66.7%)	23/35 (65.7%)
	**Loss**	2/17 (11.8%)	6/18 (33.3%)	12/35 (34.3%)

Table S2. Digital pathology-derived quantification of proliferative and p53-associated biomarkers according to methylation-defined tumor subgroup

Variable	Methylation Negative LGUC	Methylation Positive LGUC	HGUC
**Ki67-positive cells (%)[a,b,c]**	4.3 (2.0–6.3)	12.2 (4.6–20.0)	24.7 (23.3–44.4)
**Mean area of Ki67-positive nuclei (μm²)[a,b]**	39.1 (36.0–42.7)	37.8 (35.4–41.6)	54.1 (49.3–61.2)
**p53-positive cells (%)**	68.2 (53.5–77.8)	66.9 (53.2–83.8)	73.1 (28.4–82.3)
**Mean area of p53-positive nuclei (μm²)[a,b]**	32.1 (31.2–35.2)	32.9 (30.4–34.8)	55.0 (45.5–61.1)

* Values are presented as median (Q1–Q3). Comparisons across groups were performed using the Kruskal–Wallis test, followed by pairwise Wilcoxon rank-sum tests with Holm adjustment for multiple comparisons. Significant differences (p-value <0.05) in comparisons are indicated by [a] HGUC vs. Methylation-Positive LGUC, [b] HGUC vs. Methylation-Negative LGUC and [c] Methylation-Positive LGUC vs. Methylation-Negative LGUC.

Table S3. Standardized immunohistochemical protocols for biomarker evaluation using the Ventana BenchMark ULTRA system

Antibody (Cat. Number)	Host Species	Detection Kit	Depparaffination	Cell conditioning (Antigen Unmasking)	Antibody (Primary)	Counterstain	Post-Counterstain
**HER2**	Rabbit	U ultraView DAB	Selected	ULTRA CC1, 64 min, 95ºC	16 min, 36ºC	Hematoxlin, 16 min	Bluing, 8 min
**GATA3 (L50-823)**	Mouse	U Optiview DAB IHC v6	Selected	ULTRA CC1, 84 min, 95ºC	28 min, 36ºC	Hematoxlin, 16 min	Bluing, 8 min
**CK5/6 (D5-16B4)**	Mouse	U Optiview DAB IHC v6	Selected	ULTRA CC1, 124 min, 95ºC	20 min, 36ºC	Hematoxlin, 16 min	Bluing, 8 min
**Ki67 (30-9)**	Rabbit	U ultraView DAB	Selected	ULTRA CC1, 64 min, 95ºC	28 min, 36ºC	Hematoxlin, 16 min	Bluing, 8 min
**p53 (DO7)**	Mouse	U Optiview DAB IHC v6	Selected	ULTRA CC1, 85 min, 100ºC	16 min, 36ºC	Hematoxlin, 16 min	Bluing, 8 min
**MTAP**	Mouse	U Optiview DAB IHC v6	Selected	ULTRA CC1, 292 min, 100ºC	32 min, 36ºC	Hematoxlin, 16 min	Bluing, 8 min
